# Análisis de las interacciones de nirmatrelvir/ritonavir en pacientes ambulatorios en atención primaria

**DOI:** 10.23938/ASSN.1056

**Published:** 2023-12-26

**Authors:** Andrea Rodríguez Esquíroz, Lorea Sanz Álvarez, Marta Marín Marín, Patricia García González, Paula Aldave Cobos, Javier Garjón Parra

**Affiliations:** Servicio Navarro de Salud-Osasunbidea Servicio Navarro de Salud-Osasunbidea (SNS-O) Subdirección de Farmacia y Prestaciones Pamplona Navarra Spain

**Keywords:** Antivirales, Interacciones farmacológicas, COVID-19, Seguridad del paciente, Atención primaria, Antiviral Agents, Drug Interactions, COVID-19 Drug Treatment, Patient Safety, Primary Health Care

## Abstract

**Fundamento:**

Nirmatrelvir/ritonavir es un antiviral oral con un alto potencial de producir interacciones farmacológicas. La población candidata a recibirlo es mayoritariamente vulnerable, con enfermedades crónicas y polimedicada. El objetivo es evaluar la validación farmacéutica previa a la administración del antiviral.

**Material y métodos:**

Las interacciones farmacológicas entre nirmatrelvir/ritonavir y el tratamiento habitual se consultaron en fichas técnicas y las herramientas de interacciones de UpToDate® y Universidad de Liverpool®. Se incluyeron las prescripciones validadas por un farmacéutico de atención primaria (abril/2022-abril/2023).

**Resultados:**

Se incluyeron 159 pacientes; en 83 se detectaron 168 interacciones que podían suponer un cambio en su tratamiento. Las estatinas (25,6%), anticoagulantes (10,7%) y antihipertensivos (10,7%) fueron los grupos terapéuticos más frecuentemente implicados. La suspensión (53,0%) y reducción de dosis (22,6%) fueron los cambios de tratamiento más frecuentes.

**Conclusiones:**

La revisión de potenciales interacciones farmacológicas, los ajustes posológicos y las modificaciones del tratamiento habitual del paciente han evitado potenciales toxicidades, mejorando la seguridad de nirmatrelvir/ritonavir.

## Abstract (en)

**Background:**

The oral antiviral nirmatrelvir/ritonavir interacts with a range of drugs. Candidate patients to receive this antiviral agent are usually vulnerable, multipathological and polymedicated. The objective is to evaluate the pharmaceutical validation prior to the administration of the antiviral.

**Material and methods:**

Drug-drug interactions between nirmatrelvir/ritonavir and patients’ usual treatment medications were checked in product information and in the UpToDate® and the University of Liverpool® interaction tools. We included validated prescriptions between April/2022 and April/2023 by a Primary Care pharmacist.

**Results:**

Of the 159 study patients, 168 interactions were found in 83 individuals, which may have led to changes of their usual treatment. Statins (25.6%), anticoagulants (10.7%), and antihypertensives (10.7%) were the most frequently implicated therapeutic groups. Discontinuation (53.0%) and dose reduction (22.6%) were the most common treatment changes.

**Conclusions:**

Our search of potential drug interactions and subsequent dose adjustments and modifications of the patient’s usual treatment has helped avoid potential toxicities ensuring a safe use of nirmatrelvir/ritonavir.

## INTRODUCCIÓN

Nirmatrelvir/ritonavir es un medicamento antiviral oral, autorizado para el tratamiento de la COVID-19 de gravedad media-moderada en adultos que no requieren aporte de oxígeno suplementario y tienen riesgo elevado de padecer enfermedad grave. Es la combinación de nirmatrelvir, un inhibidor de la proteasa principal del SARS-CoV-2 que bloquea su replicación, y ritonavir que potencia el efecto de nirmatrelvir, inhibe de manera potente el citocromo CYP3A4 y actúa sobre otros citocromos inhibiéndolos o induciéndolos de una manera más débil. Esto conduce a que interaccione con muchos medicamentos y, en ocasiones, tenga consecuencias graves en el paciente^1^.

La situación sanitaria en la pandemia hizo que, desde su comercialización, la Agencia Española del Medicamento y Productos Sanitarios (AEMPS) gestionara su uso según unos criterios de priorización de los pacientes de mayor riesgo de progresar a COVID-19 grave. Inicialmente, se priorizó a pacientes receptores de trasplante de progenitores hematopoyéticos, CAR-T o trasplante de órgano sólido; con inmunodeficiencias primarias, tratamientos anti CD-20 o belimumab en los meses anteriores, con fibrosis quística o síndrome de Down. Estos criterios de priorización fueron cambiando a lo largo de los primeros meses de uso, añadiendo nuevos grupos de pacientes que podrían beneficiarse del tratamiento antiviral. En febrero de 2023 desapareció la priorización, por considerar la situación epidemiológica menos compleja y por la disponibilidad de diferentes tratamientos^2^, y la prescripción pasó a ser a criterio médico, siempre y cuando cumpliera la indicación autorizada (gravedad media-moderada en adultos que no requieren aporte de oxígeno suplementario y tienen riesgo elevado de padecer enfermedad grave) y se utilizara en los 5 primeros días tras el inicio de los síntomas.

Dado el elevado potencial de interacción de nirmatrelvir/ritonavir, es necesaria una revisión cuidadosa del tratamiento de los pacientes candidatos al tratamiento, en su mayoría polimedicados y con patologías crónicas^3^^-^^5^. Por ello, la AEMPS puso de manifiesto la necesidad de garantizar la validación farmacéutica previa a la dispensación.

Por otra parte, en Navarra se consideró prioritario facilitar la accesibilidad al medicamento en los distintos niveles asistenciales, facilitando su dispensación desde las oficinas de farmacia y evitando desplazamientos a los hospitales. Para ello, se establecieron unos circuitos que implicaban a farmacéuticos de atención primaria (FAP) y de los servicios de farmacia hospitalaria, con el fin de asegurar la validación y dispensar el fármaco en el menor tiempo posible y de la forma más segura.

Este trabajo tiene como objetivo evaluar la utilidad de la validación farmacéutica, describiendo las modificaciones en el tratamiento habitual motivadas por la prescripción de nirmatrelvir/ritonavir en pacientes ambulatorios.

## MATERIAL Y MÉTODOS

Se incluyeron todas las prescripciones de nirmatrelvir/ritonavir realizadas en receta electrónica entre abril de 2022 y abril de 2023 en Navarra, validadas y autorizadas por un FAP.

La validación farmacéutica comprendió la revisión de la adecuación de la indicación a las condiciones de uso establecidas por la AEMPS en cada momento, la adecuación del tratamiento según las condiciones autorizadas en ficha técnica (contraindicación en caso de insuficiencia renal o hepática grave y ajuste de dosis en caso de insuficiencia renal moderada), y de las interacciones farmacológicas con el tratamiento habitual del paciente.

Las variables recogidas fueron: edad (años), sexo (hombre, mujer), días desde el inicio de los síntomas de la infección, número de vacunas anti-SARS-CoV-2 recibidas, anticuerpos post-vacunales (sí, no, desconocido), ajuste de dosis de nirmatrelvir/ritonavir a función renal (si, no, atendiendo al filtrado glomerular: >= 60 mL/min, no ajustar; 30-59 mL/min, reducir dosis a 150 mg/100 mg cada 12 horas; <30 mL/min, contraindicado), tratamiento habitual, interacciones farmacológicas (número de medicamentos que interaccionaban con nirmatrelvir/ritonavir) y número de modificaciones de tratamiento. Los datos demográficos y clínicos de pacientes se consultaron en la historia clínica informatizada, mientras que las interacciones se consultaron en las fichas técnicas y en las herramientas de interacciones de UpToDate®^6^ y la Universidad de Liverpool®^7^.

Las variables cualitativas se describieron con porcentajes y las cuantitativas con la media y el rango o la desviación estándar. El análisis de los datos se hizo con el programa Excel®.

Los datos fueron recogidos por los FAP encargados de la validación en el ejercicio de sus funciones, no implicando acceso a datos diferentes a los necesarios para esta, ni transferencia a otras personas. El estudio fue aprobado por el Comité de Investigación Clínica con medicamentos de Navarra (número EO/2023_09).

## RESULTADOS

Se dispensó nirmatrelvir/ritonavir a 218 pacientes a través de la receta electrónica durante el periodo de estudio. En 159 casos (72,9%) la validación la había realizado un FAP y los pacientes fueron incluidos en este estudio; el resto fueron validadas por los servicios de farmacia hospitalaria.

Los pacientes tenían 66,2±15,1 años de edad media, con un ligero predominio de varones. El 87,4% habían recibido tres o más dosis de vacuna anti-SARS-CoV-2 y el tiempo medio transcurrido desde la aparición de los síntomas de la infección hasta la prescripción del antiviral fue 2,1 días (rango: 0 a 9). El 12,6% de los pacientes recibieron la dosis ajustada a la función renal (Tabla 1).

**Tabla 1:** Características de los pacientes del estudio

Características de pacientes	n (%)
Edad (años), *media* (*DE*)	66,2 (±15,1)
Sexo (hombre)	85 (53,5)
Número de vacunas anti-SARS-CoV-2 recibidas	
0	7 (4,4)
1	0 (0)
2	13 (8,2)
3	75 (47,2)
4	60 (37,7)
>4	4 (2,5)
Anticuerpos post-vacunales	
Sí	38 (23,9)
No	15 (9,4)
Desconocido	106 (66,7)
Días desde el inicio de síntomas, *media* (*rango*)	2,1 (0-9)
Dosis ajustada a función renal	20 (12,6)

DE: desviación estándar.

Durante la validación, se detectaron interacciones farmacológicas entre nirmatrelvir/ritonavir y el tratamiento habitual en 83 pacientes (52,2%) hasta con seis medicamentos, aunque en el 70% de los casos fue con uno o dos. De las 168 interacciones detectadas, las más frecuentes fueron con estatinas (25,6%), seguidas de anticoagulantes (10,7%), antihipertensivos (10,7%), medicamentos urológicos (9,5%), analgésicos opioides (8,9%), ansiolíticos (6,5%) y tratamientos onco-hematológicos (6,5%) (Tabla 2).

**Tabla 2:** Interacciones farmacológicas detectadas entre nirmatrelvir/ritonavir y el tratamiento habitual del paciente

	n (%)
Interacción con nirmatrelvir/ritonavir	
No	76 (47,8)
Sí	83 (52,2)
1	30 (18,9)
2	28 (17,6)
3	18 (11,3)
4	5 (3,1)
5	1 (0,6)
6	1 (0,6)
Medicamento que interacciona	
Estatinas	43 (25,6)
Anticoagulantes	18 (10,7)
Antihipertensivos	18 (10,7)
Medicamentos urológicos	16 (9,5)
Analgésicos opioides	15 (8,9)
Ansiolíticos	11 (6,5)
Medicamentos onco-hematológicos	11 (6,5)
Antidepresivos	9 (5,4)
Inhaladores	4 (2,4)
Inmunosupresores	4 (2,4)
Analgésicos no opioides	4 (2,4)
Terapia cardiaca	4 (2,4)
Antidiabéticos	2 (1,2)
Antihistamínicos	2 (1,2)
Antimicrobianos	2 (1,2)
Anticonceptivos	1 (0,6)
Antiagregantes	1 (0,6)
Antieméticos	1 (0,6)
Terapia oftálmica	1 (0,6)
Recomendaciones	
Suspensión del fármaco habitual durante el tratamiento antiviral	89 (53,0)
Reducción de dosis	38 (22,6)
Monitorización de reacciones adversas a medicamentos	22 (13,1)
Sustitución por otro fármaco más seguro	6 (3,6)
Monitorización del INR	5 (3,0)
Monitorización de tensión arterial	3 (1,8)
Monitorización de niveles plasmáticos	2 (1,2)
Monitorización de glucemia	1 (0,6)
Retraso del ciclo del tratamiento oncológico	1 (0,6)
Uso de métodos de barrera en paciente con anticoncepción oral	1 (0,6)

Las recomendaciones más frecuentes fueron: suspensión del fármaco habitual durante el tratamiento antiviral (53,0%), reducción de dosis (22,6%) y monitorización de reacciones adversas a medicamentos (RAM) (13,1%). Otras propuestas realizadas se muestran en la Tabla 2. El mecanismo de interacción, el efecto en las concentraciones plasmáticas y el manejo de los diferentes medicamentos habituales se recogen en la Tabla 3. Todas las recomendaciones fueron aceptadas por los prescriptores.

**Tabla 3:** Interacciones farmacológicas de los fármacos habituales del paciente con nirmatrelvir/ritonavir: mecanismo, efecto en la concentración plasmática y ajustes de tratamiento realizados

Fármaco	Mecanismo de interacción	Efecto en concentración plasmática	Manejo del fármaco
Estatinas			
Atorvastatina	Inhibición CYP3A4	Aumento	Suspender
Rosuvastatina			
Simvastatina			
Anticoagulantes			
Apixabán	Inhibición CYP3A4 y gp-P	Aumento	Reducir dosis
Rivaroxabán			Cambio a heparina
Dabigatrán	Inhibición gp-P		Reducir dosis
Edoxabán			Monitorizar signos de sangrado
Acenocumarol	Inducción CYP2C9, CYP1A2 y CYP2C19.	Disminución	Monitorizar INR o cambio a heparina
Antihipertensivos			
Amlodipino	Inhibición CYP3A4	Aumento	Reducir 50% dosis antihipertensivo y monitorizar TA
Diltiazem			
Manidipino			
Medicamentos urológicos			
Silodosina	Inhibición CYP3A4 y gp-P	Aumento	Suspender y monitorizar TA
Tamsulosina	Inhibición CYP3A4		
Fesoterodina			
Sildenafilo			
Solifenacina			
Analgésicos opioides			
Fentanilo	Inhibición CYP3A4	Aumento	Monitorizar estrechamente RAM y reducir dosis si es necesario
Oxicodona			
Morfina	Inducción UGT1A1		
Tramadol	Inhibición CYP3A4 y CYP2D6		
Ansiolíticos			
Clonazepam	Inhibición CYP3A4	Aumento	Suspender
Diazepam			
Ketazolam			
Alprazolam			Reducir 50% dosis o monitorizar estrechamente RAM y reducir/suspender si es necesario
Zolpidem			Monitorizar estrechamente RAM y suspender si es necesario
Medicamentos onco-hematológicos			
Ibrutinib	Inhibición CYP3A4 y CYP2D6	Aumento	Reducir dosis o suspender
Ciclofosfamida	Inhibición CYP3A4		Monitorizar RAM
Ruxolitinib			Suspender
Venetoclax			
Deferasirox			
Pazopanib			
Palbociclib			Retrasar inicio del ciclo
Antidepresivos			
Trazodona	Inhibición CYP3A4	Aumento	Reducir 50% dosis
Paroxetina	Inhibición CYP3A4 y CYP2D6		Monitorizar RAM
Mirtazapina			
Inhaladores			
Fluticasona	Inhibición CYP3A4	Aumento	Suspender
Salmeterol			
Inmunosupresores			
Everolimus	Inhibición CYP3A4	Aumento	Suspender
Tacrolimus			Suspender o monitorizar Cp y reajustar dosis
Analgésicos no opioides			
Metamizol	Inducción CYP3A4, inhibición CYP2B6	Aumento (y disminución Cp nirmatrelvir/ ritonavir)	Suspender. Si dolor/fiebre, utilizar paracetamol
Terapia cardiaca			
Digoxina	Inhibición gp-P	Aumento	Monitorizar digoxinemia y ajustar dosis
Sacubitrilo/valsartán	Inhibición OATP1B1		Monitorizar TA
Eplerenona	Inhibición CYP3A4		Suspender
Ivabradina			
Antidiabéticos			
Insulina	Inhibición CYP3A4	Aumento	Monitorizar glucemias y ajustar dosis
Repaglinida			Suspender
Antihistamínicos			
Hidroxizina	Inhibición CYP3A4	Aumento	Monitorizar RAM y suspender si aparecen
Loratadina	Inhibición CYP3A4 y CYP2D6		Suspender
Antimicrobianos			
Isavuconazol	Inhibición CYP3A4/5 y UGTs	Aumento	Suspender
Anticonceptivos			
Desogestrel/etinilestradiol	Inducción CYP2C9 y CYP2C19	Disminución	Utilizar métodos de barrera
Antiagregantes			
Clopidogrel	Inducción CYP2C19, CYP1A2, CYP2B6	Disminución	Cambiar a ácido acetilsalicílico
Antieméticos			
Domperidona	Inhibición CYP3A4	Aumento	Suspender
Terapia oftálmica			
Brinzolamida	Inhibición CYP3A4	Aumento	Monitorizar RAM

Cp: concentraciones plasmáticas; gp-P: glicoproteína P; RAM: reacciones adversas a medicamentos; TA: tensión arterial.

## DISCUSIÓN

En nuestro estudio, la mitad de los pacientes estaba en riesgo de que la administración de nirmatrelvir/ritonavir provocara interacciones con su tratamiento habitual. Al tratarse de un medicamento nuevo, la mayoría de las interacciones son de carácter teórico, basadas en las interacciones farmacológicas con otros inhibidores potentes del CYP3A4 o de ritonavir en otras combinaciones. En algunos casos, aunque la potencial interacción no fuese grave, el clínico decidió suspender el tratamiento crónico para evitar posibles complicaciones. Algunos ejemplos fueron: loratadina, salbutamol, deferasirox, fesoterodina o palbociclib. Como se ha publicado, aunque las recomendaciones de modificación de tratamiento son generales, el prescriptor debe individualizar el manejo de cada paciente teniendo en cuenta sus características características^8^.

Hay varios estudios publicados que proporcionan recomendaciones sobre el manejo del tratamiento ambulatorio durante el uso de este medicamento, algunas generales^5^^,^^8^^,^^9^ y otras de grupos terapéuticos específicos como medicamentos cardiovasculares^4^. Sin embargo, pocos estudios evalúan el cambio realizado en el tratamiento habitual del paciente durante la prescripción de nirmatrelvir/ritonavir.

Un estudio realizado en 5.698 pacientes ancianos (78 años de media) y polimedicados (10 tratamientos concomitantes de media) mostró que el 67,9% recibían al menos un fármaco que interaccionaba con nirmatrelvir/ritonavir, siendo los más frecuentes los medicamentos antitrombóticos (37,4%) y las estatinas (33,4%). Concluyó que una desprescripción previa al tratamiento antiviral podría aumentar el número de pacientes candidatos de manera segura^10^. Las interacciones detectadas fueron superiores que en nuestro estudio, lo que podría ser debido, en parte, a la diferencia de edad entre los dos estudios. Nuestra población es más joven, lo que podría justificar una menor polimedicación y como consecuencia un menor riesgo de sufrir interacciones farmacológicas. La mayor proporción interacciones con medicamentos antitrombóticos en nuestro estudio también podría estar justificada por la edad.

Un estudio danés corrobora el aumento de interacciones farmacológicas a medida que avanza la edad. Concluyen que el riesgo de interacciones farmacológicas relevantes se relacionan con las características de la población, ya que el 20% de pacientes mayores de 65 años y el 30% de pacientes mayores de 80 estaban en tratamiento con anticoagulantes orales, y un 15-18% de la población con estatinas, por lo que las potenciales interacciones con nirmatrelvir/ritonavir podían ser frecuentes. Por otra parte, concluyeron que el antiviral interacciona con otros fármacos de uso habitual como analgésicos, bloqueantes de canales de calcio o digoxina, lo que sugiere la importancia de la revisión del tratamiento de los pacientes previo a su uso, especialmente en personas mayores^11^.

Otro estudio en 68 pacientes identificó 101 interacciones farmacológicas, 60% de las cuales requirieron modificar el tratamiento habitual. En nuestro trabajo, las modificaciones del tratamiento fueron superiores (entorno a un 80% teniendo en cuenta suspensiones de tratamiento, reducciones de dosis y sustituciones por una alternativa terapéutica segura). Además, en otro 20% de las interacciones, las modificaciones de dosis estaban supeditadas a la monitorización de reacciones adversas o eficacia (tensión arterial, INR, niveles plasmáticos del fármaco o glucemias). En cuanto a los medicamentos implicados, las interacciones con estatinas fueron también las más frecuentes, seguidas de antihipertensivos y antidepresivos^12^.

De estos estudios se deduce que detectar previamente a la dispensación las posibles interacciones farmacológicas asegura un correcto uso de nirmatrelvir/ritonavir y minimiza las RAM. Como se observó en una serie de 72 pacientes en tratamiento con anticoagulantes orales y nirmatrelvir/ritonavir que siguieron las mismas recomendaciones que en nuestro estudio, los eventos de sangrado y tromboembólicos en el mes posterior al tratamiento se redujeron a tres: dos sangrados anales menores y uno vaginal^13^.

La principal limitación del trabajo es que se incluyeron pacientes a los que se dispensó el tratamiento en las oficinas de farmacia y no a los que se validó y/o dispensó en los servicios de farmacia hospitalaria. Tampoco se incluyeron pacientes a los que no se les autorizó el tratamiento por contraindicación, interacción grave o por no cumplir los criterios de priorización.

En conclusión, el papel del farmacéutico de atención primaria en la validación farmacéutica previa a la dispensación de nirmatrelvir/ritonavir es imprescindible y decisivo para asegurar que los criterios establecidos por la AEMPS se cumplían y para evitar potenciales toxicidades al revisar interacciones con el tratamiento habitual y modificar los tratamientos crónicos. Todo ello ha contribuido a mejorar la seguridad de uso de nirmatrelvir/ritonavir.
